# Outpatient Phenibut Taper Supported by Baclofen and Gabapentin

**DOI:** 10.7759/cureus.110132

**Published:** 2026-06-02

**Authors:** Paul Simon, Jason Yost

**Affiliations:** 1 Family Medicine, Providence St. Joseph Hospital Eureka, Eureka, USA

**Keywords:** baclofen-assisted taper, outpatient detoxification, phenibut dependence, phenibut withdrawal, substance use disorder management

## Abstract

Phenibut (β-phenyl-γ-aminobutyric acid) is a non-regulated gamma-aminobutyric acid (GABA)ergic compound associated with dependence and a clinically significant withdrawal syndrome characterized by anxiety, insomnia, and autonomic symptoms. Evidence-based guidance for phenibut withdrawal management is limited. We describe a 27-year-old male with phenibut dependence who achieved cessation in an outpatient setting using a gradual phenibut taper supported by baclofen and adjunctive gabapentin. Phenibut was tapered from approximately 2.5 g/day to cessation over nine weeks. Baclofen and gabapentin were introduced as adjunctive agents during the taper and subsequently discontinued. The patient's scheduled left shoulder labral repair surgery served as a significant external motivator for cessation. This case illustrates the feasibility of a clinician-guided outpatient taper strategy incorporating pharmacologic support in a motivated patient, while highlighting important limitations related to attribution of treatment effects and follow-up.

## Introduction

Phenibut (β-phenyl-γ-aminobutyric acid) is a central nervous system depressant with primary action at gamma-aminobutyric acid (GABA)-B receptors and secondary effects on voltage-gated calcium channels [1]. Although not approved by the U.S. Food and Drug Administration as a dietary supplement, phenibut is legal to possess, remains widely available online, and is commonly marketed for anxiety, sleep, and cognitive enhancement [1,2].

Chronic phenibut use can lead to tolerance and a withdrawal syndrome marked by severe anxiety, insomnia, tremor, dysphoria, and, in some cases, psychosis or autonomic instability [3-5]. Because phenibut use is generally outside medical supervision, clinicians may lack accurate dosing histories or standardized management protocols. Abrupt cessation has been associated with severe symptoms, prompting the use of gradual tapering and pharmacologic substitution strategies extrapolated from alcohol and benzodiazepine withdrawal management [6].

Baclofen, a prescription GABA-B receptor agonist, is structurally and pharmacologically related to phenibut and has been described in isolated case reports as a potential agent to mitigate withdrawal symptoms [3,5]. However, clinical data remain sparse, and optimal dosing strategies are not established. This report describes an outpatient phenibut taper supported by baclofen and adjunctive gabapentin, emphasizing feasibility, patient motivation, and shared decision-making.

## Case presentation

A 27-year-old male undergraduate student with a history of kratom dependence and childhood asthma presented seeking assistance with phenibut dependence. He reported using phenibut hydrochloride powder purchased online, measured with a digital scale, for the management of anxiety symptoms. He did not carry a formal anxiety disorder diagnosis and was not under psychiatric care at baseline. His peak daily use was estimated at 8-10 g/day, with a current intake of approximately 2.0-2.5 g/day at presentation. Missed doses produced withdrawal symptoms within 24 hours, including anxiety, insomnia, lethargy, dysphoria, and a subjective sense of impending doom.

The patient had previously tapered kratom successfully using gabapentin, though the details and timeline of kratom cessation were unavailable. He denied alcohol use disorder, opioid use disorder aside from kratom, benzodiazepine dependence, or intravenous drug use.

Initial assessment and planning

Inpatient medically supervised detoxification was initially recommended for medical monitoring, but was not feasible due to financial constraints. An outpatient approach was pursued using shared decision-making. Gabapentin 300 mg three times daily was initially prescribed to assist with managing withdrawal symptoms due to prior benefit in tapering kratom, also a dependence-causing substance, but the dosing was reduced to nightly administration due to sedation.

After discussion of limited published literature, including prior case reports describing baclofen-supported withdrawal, a shared decision was made to initiate a gradual phenibut taper supported by baclofen. The patient was counseled regarding the lack of standardized protocols, the off-label nature of baclofen use, the importance of close follow-up, and explicit counseling that efficacy was unproven and that gradual phenibut tapering alone may have been sufficient.

Taper strategy and clinical course

At presentation, the patient reported current phenibut intake of approximately 2.0-2.5 g/day and withdrawal symptoms within 24 hours of missed doses. Table 1 summarizes the outpatient taper schedule and adjunctive pharmacotherapy with clinical timeline and dosing decisions. Phenibut was tapered gradually over approximately nine weeks, with dose reductions guided by symptom tolerance. Baclofen was initiated early in the taper and titrated cautiously due to a lack of a standardized protocol. Dosing was based on clinical judgment and review of a case study that suggested treating with 10 mg of baclofen for every 1 gram of phenibut [3]. Gabapentin was maintained only at night and tapered first to reduce the risk of secondary dependence. The patient’s planned left shoulder labral repair surgery became a strong motivator for cessation due to concerns about perioperative withdrawal and anesthesia safety. Phenibut was discontinued prior to surgery, followed by the gradual elimination of baclofen and gabapentin. Laboratory testing, including complete blood count, comprehensive metabolic panel, and thyroid-stimulating hormone, was obtained in week 12 following cessation of phenibut to ensure no metabolic abnormalities. All laboratory values were within normal reference ranges (Table 2).

**Table 1:** Summary of outpatient phenibut taper and adjunctive pharmacotherapy *Weeks 13-15 dosing based on patient self-report during telephone follow-up. Specific taper increments were not documented.

Week	Phenibut (g/day)	Baclofen (mg/day)	Gabapentin (mg/night)	Clinical notes
0	2.0-2.5	0	300	Initial visit; inpatient treatment recommended but not feasible due to financial constraints. Gabapentin initiated
1	2.0	5	300	Baclofen initiated. Planned phenibut taper of 0.5 g/week
2	1.8	10	300	Patient reduced taper to 0.2 g/1-2 weeks due to withdrawal symptoms
3	1.4	15	300	Patient tolerating taper. Withdrawal symptoms manageable
6	1.0	15	300	Phenibut nearing cessation. Residual insomnia and anxiety lingers
8	0.5	15	300	Phenibut nearly discontinued. Patient motivated to quit prior to the upcoming left shoulder labral repair surgery. Shared decision-making to maintain the current baclofen dose
9	0	15	300	Patient successfully tapered off phenibut three days prior to labral repair surgery. Withdrawal symptoms present but tolerable. Behavioral support started with a school counselor
12	0	10	200	Baclofen and gabapentin taper initiated. Patient remains abstinent from phenibut. Blood work obtained
13-15*	0	0	0	Patient self-completed tapers and continued phenibut abstinence (self-report)

**Table 2:** Laboratory results obtained after phenibut cessation

Test category	Analyte	Result	Reference range
Complete blood count (CBC)	White blood cell count (WBC)	3.8×10³/µL	3.4-10.8×10³/µL
	Hemoglobin (Hgb)	15.3 g/dL	13.0-17.7 g/dL
	Platelet count (Plt)	276×10³/µL	150-450×10³/µL
Comprehensive metabolic panel (CMP)	Sodium (Na)	138 mmol/L	135-145 mmol/L
	Potassium (K)	4.1 mmol/L	3.5-5.0 mmol/L
	Creatinine (Cr)	1.03 mg/dL	0.76-1.27 mg/dL
	Alanine aminotransferase (ALT)	24 IU/L	0-44 IU/L
	Aspartate aminotransferase (AST)	21 IU/L	0-40 IU/L
Endocrine	Thyroid-stimulating hormone (TSH)	1.28 µIU/mL	0.45-4.5 µIU/mL

Outcome and follow‑up

The patient did not return for in-person follow-up after week 12 but was contacted by phone. The patient reported sustained abstinence from phenibut and complete discontinuation of both baclofen and gabapentin. He reported an uncomplicated perioperative course following shoulder labral repair and endorsed minimal residual anxiety with intermittent sleep disturbance that did not impair functioning. Abstinence and symptom improvement were based on self-report without biochemical confirmation.

## Discussion

This case illustrates the feasibility of a clinician-guided outpatient phenibut taper supported by baclofen with adjunctive gabapentin in a motivated patient. The pharmacologic rationale for baclofen use lies in its GABA-B receptor agonism, which closely mirrors the primary mechanism of phenibut. Phenibut itself is a GABA analog that exerts its effects predominantly through GABA-B receptor agonism, with additional influence on GABA-A receptors and voltage-gated calcium channels. This mechanistic overlap provides a theoretical basis for substitution strategies using baclofen to mitigate withdrawal symptoms [1,6].

Gabapentin was used adjunctively in this case, primarily to address insomnia and anxiety symptoms. While its specific role in phenibut withdrawal remains unclear, gabapentin has been studied more extensively in alcohol withdrawal and other substance use disorders. Evidence suggests that gabapentin can modulate GABAergic and glutamatergic neurotransmission through effects on voltage-gated calcium channels, thereby reducing withdrawal symptoms such as anxiety, insomnia, and dysphoria. Meta-analyses and clinical studies indicate that gabapentin may be effective in reducing withdrawal severity and promoting abstinence in alcohol use disorder, though results remain mixed and context-dependent [7,8]. Its role in this case should therefore be interpreted as supportive rather than definitive.

Several factors limit interpretation. First, causality cannot be attributed to baclofen or gabapentin independently, as multiple interventions were used concurrently, including gradual phenibut tapering, adjunctive gabapentin, and behavioral counseling. Published literature similarly emphasizes that phenibut withdrawal management often requires multimodal therapy, making it difficult to isolate the effects of individual agents [9]. Gradual tapering and heightened motivation related to an upcoming surgical procedure may have been sufficient. Second, withdrawal severity was assessed clinically rather than using validated symptom scales. Third, follow-up after phenibut cessation relied on patient self-report without in-person assessment or confirmation.

Notably, the patient’s planned orthopedic surgery functioned as a powerful external motivator. Anticipated anesthesia exposure, perioperative safety concerns, and postoperative recovery appeared to enhance adherence and engagement. Behavioral science literature describes such “teachable moments” as opportunities in which acute health-related events increase patient motivation to modify risky behaviors. While not specific to phenibut use, this concept has been observed across multiple domains of substance use and perioperative care, suggesting that time-limited medical events may be leveraged therapeutically and enhance adherence to treatment plans [10].

An important feature of this case is the outpatient management approach. While inpatient detoxification remains the standard for patients at high risk of severe withdrawal, emerging case-based evidence suggests that outpatient tapering may be appropriate for carefully selected individuals with stable social circumstances and reliable follow-up. However, clinicians must remain vigilant given the unpredictable course of withdrawal and the potential for rapid clinical deterioration [9].

Limitations

This case report is limited by its single-patient design, lack of standardized withdrawal measures, incomplete in-person follow-up, and reliance on retrospective self-reported abstinence. The simultaneous use of multiple interventions precludes conclusions regarding the efficacy or safety of any individual component.

## Conclusions

This case demonstrates the feasibility of an outpatient, patient-centered phenibut taper supported by baclofen and adjunctive gabapentin in a motivated individual. While baclofen may be a useful adjunct in managing phenibut withdrawal, further controlled studies are needed to establish standardized treatment protocols and clarify the roles of baclofen, gabapentin, and other adjunctive therapies in phenibut withdrawal management. Clinicians should employ cautious, individualized approaches and consider leveraging salient medical events as behavioral motivators for cessation.
